# Malaria epidemics in Colombia, 1970-2019

**DOI:** 10.1590/0037-8682-0559-2021

**Published:** 2022-04-29

**Authors:** Julio Cesar Padilla Rodríguez, Mario Javier Olivera, María Cristina Padilla Herrera, Edwin Pachón Abril

**Affiliations:** 1 Red Gestión de Conocimiento, Ivestigación e Innovación en Malaria, Bogotá, Colombia.; 2 Instituto Nacional de Salud, Grupo de Parasitología, Bogotá, Colombia.; 3 Ministerio de Salud y Protección Social, Dirección de Epidemiología y Demografía, Bogotá, Colombia.

**Keywords:** Malaria, Epidemiology, Epidemics, Colombia, Public health surveillance

## Abstract

**Background:**

Malaria has unstable transmission in Colombia and has variable endemic-epidemic patterns. This study describes the epidemiological characteristics of malaria epidemics registered in Colombia from 1970-2019.

**Methods:**

Data from 1979-2019 were collected from the National Public Health Surveillance System. The data was tabulated and pertinent descriptive analyses were carried out.

**Results:**

Fifteen malaria outbreaks and approximately five-year-long epidemic cycles were observed in Colombia during the study period.

**Conclusions:**

Malaria epidemics in Colombia present a five-yearly transmission pattern, mainly due to the increased vulnerability produced by seasonal population migrations in receptive areas with active transmission.

The World Health Organization considers the reduced transmission in the last two decades to be one of the unprecedented successes in the fight against malaria, with 1.5 billion cases averted and 7.6 million lives saved globally^1^. The incidence of malaria decreased from 81 in 2000 to 59 per 1,000 population at risk in 2019, before increasing again to 59 per 1,000 in 2020. This increase in 2020 was associated with the disruption of one or more malaria-related services during the COVID-19 pandemic. Ninety-six percent of malaria cases worldwide are accounted for by 29 African countries with stable transmission, including Nigeria (27%), the Democratic Republic of the Congo (12%), Uganda (5%), Mozambique (4%), and Angola (3.4%). In these countries, transmission is high, without marked fluctuations during the year; moreover, the population is highly immune, and epidemics are unlikely. Transmission is almost perennial; it is hardly affected by climatic changes, it is caused by highly infective vectors, and it presents a high prevalence of *Plasmodium falciparum* ^2^.

In contrast, in the Americas, transmission is unstable, of moderate-to-low intensity, and has yearly variation. The collective immunity of the population is low, and epidemics, caused by climatic changes and environmental alterations produced by humans in receptive transmission scenarios, are frequent^3^. In this region, there has been a significant reduction in malaria cases, from 1.5 million to 0.65 million cases (58% reduction) and a reduction in associated deaths from 909 to 409 cases in the last two decades^1^. Seventy-seven percent of the malaria burden in the region was reported in Brazil, the Bolivarian Republic of Venezuela, and Colombia^1^.

In Colombia, approximately 70,000 cases are registered annually, with *P. vivax* infection predominating^4^. Colombia has also presented a marked reduction in the incidence of the disease (approximately 40%); however, the transmission scenario is currently characterized by a higher frequency of epidemic outbreaks caused by intense migration, illegal mining, illicit crop cultivation, and armed social conflict^5^.

This new epidemiological scenario plays an important role in the design and implementation of malaria elimination plans in national territories. However, it has not been considered yet, and areas prone to epidemics need to be identified based on the study of historical epidemics and the local determinants that influence the increase in vulnerability favoring the appearance of epidemics^6^ ^,^ ^7^. This study aimed to identify the epidemiological characteristics of malaria epidemics registered in Colombia between 1970 and 2019.

This descriptive and retrospective observational study was conducted on malaria epidemics that occurred in Colombia between 1970 and 2019. Variables of place, time, and people were defined, and data were extracted from official sources of secondary information provided by the National Malaria Program, Directorate of Demography and Epidemiology of the Ministry of Health, and National Public Health Surveillance System.

The years with case numbers greater than the expected average number of cases in each corresponding decade in the period studied were considered as epidemic years, conceptualized as the excess morbidity observed.

The following classification of the eco-epidemiological regions of malaria transmission in the country was adopted: 1) Pacific (Cauca, Choco, Nariño, and Valle departments); 2) Uraba-Bajo Cauca-Sinu-San Jorge (departments of Antioquia and Córdoba); 3) Amazonia (departments of Amazonas, Caqueta, Guaviare, Guainia, Putumayo, and Vaupés); 4) Orinoquia (departments of Arauca, Meta, Casanare and Vichada); 5) Caribbean (departments of Atlantico, Bolivar, Cesar, La Guajira, Magdalena, San Andres, and Sucre); and 6) Andean (departments of Boyaca, Cundinamarca, Huila, Tolima, Norte de Santander, Santander, Caldas, Quindío, and Risaralda).

The data were collected in a database designed in MS Excel (Microsoft, Redmond, USA) and analyzed using Stata (release 15, Stata Corporation, College Station, TX, USA), whereas ArcGIS version 10.5 (ESRI, Redlands, CA) was used to produce maps. Absolute measures were used, such as the number of cases, and relative measures, such as proportions, annual parasitic index (API × 1,000 population at risk), and lethality × 100. For the eco-epidemiological regions, the median API of the corresponding epidemic period was used. Descriptive analyses were performed using measures of central tendency such as means and medians. With this information, the distribution of outbreaks was mapped by region to identify foci of active and persistent transmission and those of seasonal and sporadic transmission. For the difference in medians between the APIs of the eco-epidemiological regions, the Mann-Whitney U test was used. P values less than 0.05 were considered statistically significant. The present study met the ethical requirements established in Resolution 8430 of 1993 of the Ministry of Health of Colombia, Article 11, which establishes that studies such as the present one are risk-free and do not require approval by the Ethics Committee. The confidentiality and anonymity of the data were guaranteed.

Fifteen outbreaks of malaria were observed in Colombia during the study period. Of these, nine (60%) occurred from 1970-1999 and in the 21^st^ century, there were six (40%) were epidemic events. The frequency or cyclicality of these events showed approximately a five-yearly behavior. By decade, there were four epidemic outbreaks in the 1990s, three outbreaks in each decade in the 1970s, 2000s, and the 2010s, and two outbreaks in the 1980s. The most intense epidemic outbreak occurred in 1998, when 258,845 cases were registered (Figure 1). The accumulated number of cases registered in the epidemic years was 2,004,049, but only 38.6% of these 773,107 cases accounted for the excess morbidity caused by the epidemics.

**FIGURE 1:**
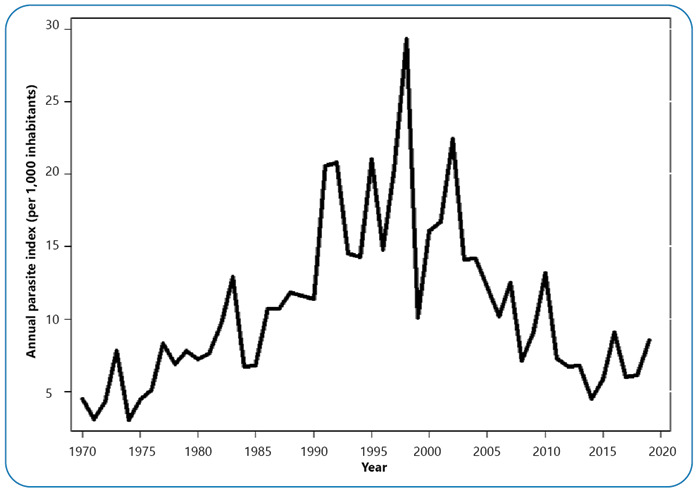
Malaria epidemics in Colombia, 1970-2019.

The median value of the excess morbidity recorded during the epidemics that occurred in the period 1970-1999 was 32.6% (maximum value 59.6% and minimum value 27.3%), and that observed during the outbreaks that occurred in the two decades of the 21^st^ century was 33.4% (maximum value 50.1% and minimum value 17, 4%). In all the epidemics, *P. vivax* and *P. falciparum* cases were reported; however, infections caused by *P. vivax* were predominant in 70% of the epidemics. However, in the epidemic outbreaks registered in 1973, 1998, 2016, and 2019, *P. falciparum* infections prevailed (Table 1).

During the epidemics, the most vulnerable population groups, corresponding to the economically active population aged 15-44 years, were most affected (Table 1). The highest excess epidemic morbidity was observed in the eco-epidemiological region of Uraba-Bajo Cauca-Sinu-San Jorge (53.4%) (median API during the epidemic period was 29.9 per 1,000), followed by the Pacific Region (32.4%) (median API during the epidemic period was 17.6 per 1,000), with a statistically significant difference (p < 0.01). These regions contributed to the cases in all the years of the epidemic. The Amazon region contributed irregularly to epidemic cases, and the remaining regions contributed sporadically (Figure 2). Outbreaks, with a predominance of *P. falciparum* infections, occurred predominantly in the Pacific region (Table 1).

**FIGURE 2:**
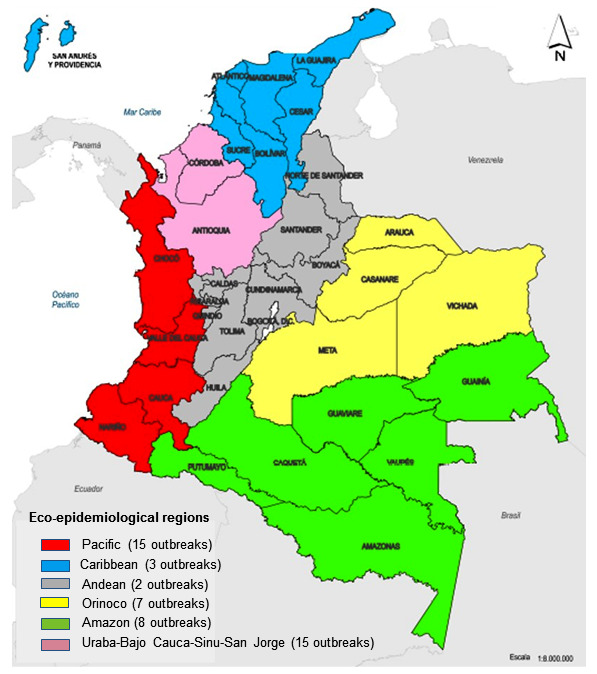
Epidemic outbreaks by eco-epidemiological regions in Colombia, 1970-2019.

During the epidemic years, cumulatively 3,998 deaths from malaria were recorded. The fatality trend recorded throughout the period showed a marked decrease, from 3.5 deaths per 100 malaria cases in 1973 to less than one death at the end of the 1990s, and it remained below 0.2 per 100 malaria cases in the first two decades of this millennium (Table 1).

**TABLE 1:** Years with malaria epidemics in Colombia, 1970-2019.

Year	Cases	Mean (Reference)	Excess morbidity					Mortality	
			N	%	*P. falciparum*	*P. vivax*	The most vulnerable age group affected (%)	N	Case fatality rate (%)
1973	56,119	25,821	30,298	54.0	18,304	11,994	15-44 (62)	1,051	3.5
1977	63,888		38,067	59.6	17,728	20,339	15-44 (53)	739	1.9
1979	60,738		34,917	57.5	13,328	21,589	15-44 (60)	526	1.5
1983	105,002	73,343	31,659	30.1	14,331	17,328	15-44 (61)	634	2.0
1988	100,850		27,507	27.3	9011	18,496	15-44 (56)	216	0.8
1991	181,156	126,038	55,118	30.4	21,264	33,854	15-44 (60)	183	0.3
1992	184,023		57,985	31.5	21,802	36,183	15-44 (63)	193	0.3
1995	187,082		61,044	32.6	20,587	40,457	15-44 (65)	76	0.1
1998	258,845		132,807	51.3	69970	62,837	15-44 (75)	258	0.2
2001	205,924	102,719	103,205	50.1	46,855	56,350	15-44 (70)	60	0.05
2002	196,148		93,429	47.6	41,389	52,040	15-44 (62)	40	0.04
2004	124,768		22,049	18.0	9,790	12,259	15-44 (60)	25	0.2
2010	117,637	64,828	52,809	44.6	14,945	37,864	15-44 (61)	23	0.04
2016	83,356		18,528	22.2	11,284	7,244	15-44 (63)	18	0.09
2019	78,513		13,685	17.4	7,299	6,386	15-44 (65)	12	0.08
**TOTAL**	**2,004,049**	**-**	**773,107**	-	**337,887**	**435,220**	**-**	**4,054**	**-**

N: number of cases.

This study shows that in Colombia, epidemic transmission registers the frequency of appearance of five-yearly epidemic cycles, mainly in endemic eco-epidemiological regions with active transmission in the national territory, and mainly affects the young and working population predominantly with *P. vivax* infections. It has been described that in unstable and low-transmission areas, when the balance between infection rates and the herd immunity of a population in a highly receptive area is affected, or when accessibility to health services is interrupted or affected, it results in epidemics^8^ ^,^ ^9^.

In the Americas, there are different epidemiological situations of malaria transmission, such as the reduction of the endemic, elimination of the disease in some countries, and re-emergence and intensification of the epidemic in the Bolivarian Republic of Venezuela^10^. Similarly, in Colombia, despite the sustained decline in endemic levels, seasonal epidemic outbreaks continue to be recorded in important regions^11^. The main determinants that alter receptivity, vulnerability, and infectivity and favor epidemic dynamics are the intense internal migration of susceptible civil, military, and illegal populations and carriers of malaria parasites^12^. This is caused by the high labor demand in enclaves of intensive illegal mining and illicit crops, which cause environmental deterioration in jungle areas with active endemic transmission, such as the Pacific region and the Uraba-Bajo Cauca-Sinu-San Jorge region. In addition, deficiencies and the precarious development of health services, climatic variations, and natural disasters lead to outbreaks^12^ ^,^ ^13^.

Despite maintaining a trend in the reduction of malaria mortality in the country during inter-epidemic periods, it suffers concomitant increases with epidemic situations. The latter is caused by problems in accessibility to timely diagnosis and effective and safe treatments for illegal, itinerant, and migrant populations during these types of situations^12^ ^,^ ^14^.

Considering this, elimination plans in the country should be implemented on priority to monitor the changes in receptivity and vulnerability; to detect, diagnose, treat and investigate cases; and, to promote the timely detection and control of outbreaks in all endemic areas of the national territory. To achieve this, the capacity of the national epidemiological surveillance system to detect an unusual increase in cases early, based on the stratification of the risk of transmission in areas of high epidemic risk in the country, must be maximized^6^ ^,^ ^15^.

In conclusion, malaria epidemics in Colombia present a five-yearly transmission pattern, mainly due to the increased vulnerability produced by seasonal population migrations in receptive areas with active transmission.
